# Correlation between Discharged Worms and Fecal Egg Counts in Human Clonorchiasis

**DOI:** 10.1371/journal.pntd.0001339

**Published:** 2011-10-04

**Authors:** Jae-Hwan Kim, Min-Ho Choi, Young Mee Bae, Jin-Kyoung Oh, Min Kyung Lim, Sung-Tae Hong

**Affiliations:** 1 Department of Parasitology and Tropical Medicine and Institute of Endemic Diseases, Seoul National University College of Medicine, Seoul, Republic of Korea; 2 National Cancer Control Institute, National Cancer Center, Goyang-si Gyeonggi-do, Republic of Korea; Asahikawa Medical College, Japan

## Abstract

**Background:**

Stool examination by counting eggs per gram of feces (EPGs) is the best method to estimate worm burden of *Clonorchis sinensis* in infected humans. The present study investigated a correlation between EPGs and worm burden in human clonorchiasis.

**Methods and Findings:**

A total of 60 residents, 50 egg-positive and 10 egg-negative, in Sancheong-gun, Korea, participated in this worm collection trial in 2006–2009. They were diagnosed by egg positivity in feces using the Kato-Katz method. After administration of praziquantel, they were purged with cathartics on the next day, and then discharged adult worms were collected from their feces. Their EPGs ranged from 0 to 65,544. Adult worms of *C. sinensis* were collected from 17 egg-positive cases, and the number of worms ranged from 1 to 114 in each individual. A positive correlation between EPGs and numbers of worms was demonstrated (*r* = 0.681, *P*<0.001). Worm recovery rates were 9.7% in cases of EPGs 1–1,000 and 73.7% in those of EPGs over 1,000. No worms were detected from egg-negative subjects. Maximum egg count per worm per day was roughly estimated 3,770 in a subject with EPGs 2,664 and 106 collected worms.

**Conclusions:**

The numbers of the worms are significantly correlated with the egg counts in human clonorchiasis. It is estimated that at least 110 worms are infected in a human body with EPGs around 3,000, and egg productivity of a worm per day is around 4,000.

## Author Summary

Clonorchiasis is a major neglected disease in East Asia. Worm data in infected humans are very limited, and only egg counts roughly estimate infection burden of the worms. In endemic areas, we recruited infected cases and tried to collect the adult worms from them. They were treated with 3 doses of praziquantel, and purged next day under fasting. Adult worms of *C. sinensis* were recovered from their diarrheal feces. The worms discharged from humans after treatment are minimum confirmed numbers. The worm recovery rate noticeably increased in subjects with higher egg counts. The number of collected worms was well-correlated with the egg counts. Worm collection by praziquantel medication and purgation is a safe non-invasive method to get worm information from human. The present study confirms that at least 110 worms are infected in a human body with egg counts per gram of feces around 3,000, and egg productivity of a worm per day is around 4,000.

## Introduction

*Clonorchis sinensis* is the most widespread parasitic fluke in East Asia. Several hyperendemic foci are scattered in China and Korea [1], [2]. Clonorchiasis, a disease caused by infection of *C. sinensis*, results from ingestion of its metacercariae by consumption of raw or undercooked freshwater fish. The metacercariae excyst in the duodenum, and the larvae migrate into the bile duct and grow up to adult worms. Adult worms produce excretory and secretory proteins, sperms, and eggs at 3 to 4 weeks after infection. It was reported that the secretory and excretory proteins are responsible for development of several pathological changes in the bile duct including cholangiocarcinoma and other complications [3].

Despite several pathological changes, most patients with clonorchiasis complain no or mild non-specific symptoms. In this respect, most infected people hardly notice this infection for a long time. Therefore an accurate diagnosis and subsequent cure in early stage of infection is essential to prevent complications caused by *C. sinensis* infection. Several kinds of methods are generally used for diagnosis of clonorchiasis, such as fecal examination by the Kato-Katz method (KK), formalin-ether sedimentation (FES), serology by enzyme-linked immunosorbent assay (ELISA), and ultrasonography of the liver [4]–[7]. Of these methods, the KK is a quantitative examination by counting the number of eggs discharged in feces.

The number of eggs per gram of feces (EPGs) has been used to conjecture the worm burden in clonorchiasis [6]. In human opisthorchiasis, adult worms were recovered after praziquantel treatment, and relationship between worm burden and EPGs was analyzed in a previous report [8]. It was known that the number of eggs per worm per day in guinea pigs and cats infected with *C. sinensis* were 1,600 and 2,400, respectively [9]. Even though we expect that EPGs per worm or egg productivity of a worm per day would be different in humans as compared with experimental animals due to differences in amounts of feces or host susceptibility, no data of infected worms have been matched with the EPGs in human clonorchiasis.

In our previous report, we recovered adult worms of *C. sinensis* from 8 patients with high EPGs [10]. The report described details of procedures for collection of *C. sinensis* adult worms from infected people and suggested its use for evaluation of human infection intensity. Collection and counting of worms should be a direct method of quantitative diagnosis. The present study followed the protocol subjecting 42 new egg-positive and 10 egg-negative individuals in an endemic area of *C. sinensis*. The worms collected in the present study and those from the 8 cases of our previous study [10] were used for the present analysis of correlation between EPG counts and the number of worms from humans.

## Materials and Methods

### Ethics statement

The present study was a part of the cohort research which was reviewed and approved by the institutional review board of the National Cancer Center of Korea in 2006 (NCCNCS-07–080), and informed consent was obtained from the subjected residents.

### Study population and location

A cohort study has investigated risk factors and incidence of cholangiocarcinoma since 2006 in Sancheong-gun, Gyeongsangnam-do, Korea, where clonorchiasis is endemic. The cohort study team of the National Cancer Center of Korea and the parasitology laboratory members of the Seoul National University collaborated for the cohort. About 500 to 700 residents in Sancheong-gun were examined every year for their general health status and parasitological screening on voluntary base. The residents who joined the health examination were included for the cohort. The present study subjected 50 egg positive and 10 egg negative residents from the cohort participants for the worm collection. The Kato-Katz method was used for quantitative stool examination, and duplicate slides per a subject were applied to reduce diagnostic variation [11].

### Worm collection

All participants, regardless of egg positivity, took 25 mg/kg praziquantel, three times a day. On the next day of praziquantel treatment, they ingested purgation solution (Colyte®-F, Taejoon Pharm., Seoul, Korea) to pass out their intestinal content. Whole purged fecal materials from the participants were collected 5 to 10 times and the worms were carefully recovered with naked eyes after washing of the feces. We followed the procedure which was described by Shen et al. [10].

### Statistical analysis

Spearman rank correlation analysis was used to evaluate the correlation between egg counts and number of recovered worms. Statistical differences between categorical variables were calculated with a Student's t or 2×2 chi-square test. All analyses were carried out with PASW Statistics, version 18.0 (SPSS Inc., Chicago, IL, USA). A *P* value of less than 0.05 was considered to indicate statistical significance.

## Results

In a total of 60 residents, 52 of the present study (42 egg-positive and 10 egg-negative) and 8 of previous report [10] were newly enrolled in this experiment. They comprised 42 males and 18 females with a median age of 58 years (range; 45–77). All of egg-positive subjects were diagnosed by the KK method. The mean EPG count of 50 positive subjects was 2,878 (range; 12–65,544).

**Figure 1:**
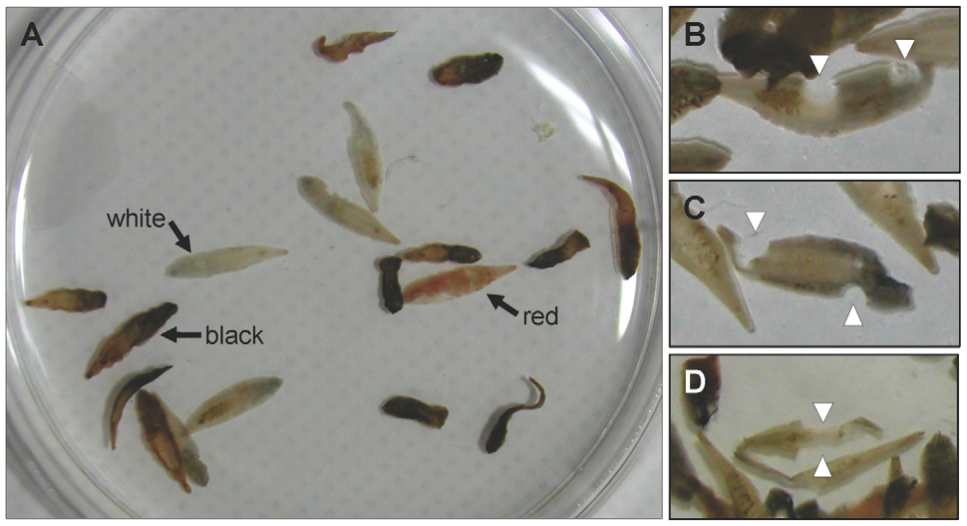
Adult worms of *C. sinensis* recovered from patients. (A) Three kinds of worms with different colors were confirmed. (B, C and D) A few worms were collected with partial maceration (arrowheads) from different subjects.

A total of 460 adult worms of *C. sinensis* were collected from 17 of the 50 egg-positive subjects. The number of worms in each subject ranged from 1 to 114. No worms were detected from egg-negative subjects (n = 10). The recovered worms were fully matured (15–20×2–4 mm in size), and their body color was red, black, or white (Fig. 1A). In black worms, the posterior part of the body mainly represented black, but the rest was red. Interestingly, white worms looked destained except for the uterus which was filled with eggs. Further, a few worms presented partial or severe damage demonstrating round margin of the remaining body (Fig. 1B, C, and D).

**Figure 2:**
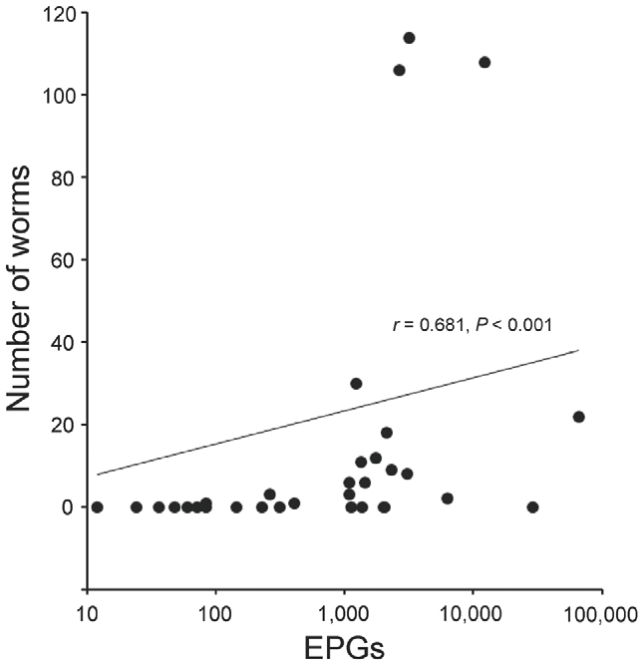
Correlation analysis between numbers of recovered worms and EPG counts. Data of egg-positive subjects (n = 50) were used for analysis. The correlation coefficient (Spearman's rho, *r*) usually presents a weak (*r*<0.5), moderate (0.5<*r*<0.8) and strong (0.8<*r*) relationship. A Student's t test was used to examine significance of the correlation coefficient and *P* value less than 0.05 was considered statistically significant.

Even though there were wide variations in the numbers of recovered worms by individuals, the numbers of worms tended to rise according to EPG counts (Table 1). When the correlation analysis was performed to evaluate quantitative changes of recovered worms with EPG counts, it revealed a positive association (*r* = 0.681, *P*<0.001) (Fig. 2).

**Table 1:** Intensities of *Clonorchis sinensis* infection by EPG ranges.

EPG range	No. of examined	Worm-positive case (%)	Worm No. range (mean)
1–100	21	1 (4.8)	1 (1.0)
101–1,000	10	2 (20.0)	1–3 (2.0)
1,001–2,000	8	6 (75.0)	3–30 (11.3)
2,001–3,000	5	3 (60.0)	9–106 (44.3)
3,001–	6	5 (83.3)	2–114 (50.8)
Total	50	17 (34.0)	1–114 (27.1)

The overall rate of worm collection was 34.0% from egg-positive subjects (Table 1). The highest rate was 83.3% in the subjects of EPGs over 3,000. The lower the EPGs were counted, the less the worms were recovered. The rate of worm collection was 75.0% in the subjects with EPGs 1,001–2,000 but decreased to 20.0% in those with EPGs 101–1,000 and 4.8% in those with EPGs 1–100 (*P* = 0.001 by Pearson's χ^2^ test).

Numbers of egg per worm per day (EPWPD) widely varied in the present results (range; 3,770–475,200). Due to the wide variation, reliable EPWPD was unable to be calculated from whole data. Therefore we focused on two subjects who discharged 114 and 106 worms; these worm numbers were higher than those of most subjects. Their EPGs were 3,168 and 2,664, and EPWPD were 4,168 and 3,770, respectively.

The rate of worm collection was little higher in males than in females, but not significant (Table 2, 43.8% versus 16.7%, *P* = 0.052 by Pearson's χ^2^ test). The overall mean EPGs were higher in males than in females, but there were wide variations in counts (2,878 versus 537, *P* = 0.246 by Student's t test). By age, the worm recovery rates decreased from 50.0% in age 40–59 to 21.4% in age 60–79 (Table 2, *P* = 0.034 by Pearson's χ^2^ test).

**Table 2:** *Clonorchis sinensis* infection by age and sex.

Age	Sex	No. of egg-positive subjects	Worm positive cases (%)
40–49	Male	7	4 (57.1)
	Female	3	1 (33.3)
	Subtotal	10	5 (50.0)
50–59	Male	10	5 (50.0)
	Female	2	1 (50.0)
	Subtotal	12	6 (50.0)
60–69	Male	10	3 (33.3)
	Female	10	1 (10.0)
	Subtotal	20	4 (20.0)
70–79	Male	5	2 (40.0)
	Female	3	0 (0.0)
	Subtotal	8	2 (25.0)
Total	Male	32	14 (43.8)
	Female	18	3 (16.7)
	Subtotal	50	17 (34.0)

## Discussion

The present study applied praziquantel medication and purgation to collect adult worms from infected human subjects of clonorchiasis as Shen et al. [10] suggested, and confirmed that the method was safe and reliable. Praziquantel is generally regarded as a safe drug for treatment of trematode and cestode infections [12], [13]. It often has side effects, such as headache, dizziness, nausea, vomiting and abdominal pain [14]. These effects are usually mild and transient, and therefore special treatment is not needed. In the present study, 25 subjects (41.7%) complained epigastric fullness, dizziness and vomiting but these symptoms subsided spontaneously on the same day. There was no such a case of anaphylactic reaction in our study as reported previously [15].

Praziquantel is known to induce paralysis by sustained musculature contraction and tegumental disruption of target worms via increase of permeability to Ca^2+^ on the cell membrane of the worms [16]. When metabolized praziquantel in bile juice contacts *C. sinensis* in the intrahepatic bile duct, the worms are paralyzed and dispatched from the bile duct mucosa [10], [17]. The paralyzed worms slowly flow down to the intestine with bile. Most of the worms recovered in the present study preserved their gross structure intact. They were rapidly expelled from the intestine by the purgation, and this process prevented them from severe destruction or autolysis. However, a few fragmented or partially disrupted worms were found but it was unable to figure out their exact numbers.

The present collection method could have recovered only the worms which moved to the intestine before the purgation. The purgation began after 12 to 15 hours from the last dose of praziquantel. The collection timing (up to 5 hours) is the maximum allowance for keeping the patients fasting and diarrhea. Adult worms of *Opisthorchis viverrini* are able to be recovered through the purgation as well, and a few were found in feces in about 12 to 24 hours after the first treatment with praziquantel [8], [18]. In the present study, it had to be considered whether a few dead worms were discharged by defecation before the purgation. We did not check their history of defecation after praziquantel administration before the purgation. This is a limitation of the present method for worm collection, but no worms were detected in the first or second stools during the purgation.

It is presumed that most of the lost worms in the collection still remain in the biliary tree, dead or alive. Most of the worms may be still in the gallbladder or bile duct because bile flow is very slow and stagnant in the gallbladder. The worms live in the intrahepatic bile duct which undergoes dilated and tortuous changes. The infected bile ducts provide saccular rooms for the worms to survive as their habitat. Some of the paralyzed worms by praziquantel may be trapped in the saccular rooms. Those trapped worms are unable to flow down to the intestine and should have been excluded in the worm collection. Further, it is possible that some worms were lost by maceration during the passage through the biliary and intestinal track. In the present study, a few worms were partially or totally damaged. The totally destroyed worms must have been lost during the collection. The round partial loss of the body tissue was recognized as an outcome of bullous rupture of the tegument, which was a known action of praziquantel [19], [20].

In the present study, the color of the worms varied with most cases. Red, black or white worms were intermingled according to individuals or in a subject. The worms whose anterior body part was red presented the uterus full of eggs. As the body color of worms is red in animals newly infected with *C. sinensis*, the red worms may be fresh and actively produce eggs. On the contrary, the white worms containing comparatively small amount of eggs in the uterus may be less productive. When the worms are pigmented by lipofuscin as a senile change, they are looking black [21]. The finding demonstrates various ages of the infected worms and continuous infection of *C. sinensis* in the studied endemic area.

The rate of worm collection was particularly low in EPGs 1–1,000 as compared with those in EPGs over 3,000. This finding means that most worms in the bile duct of the infected subjects were lost or missed during the collection process. The subjects with high EPGs were expected to be infected with many worms, however, a certain proportion of worms must have been lost even from those with high EPGs because 33 (66.0%) of the 50 egg-positive subjects were worm-negative. Also the wide variation (2–114 worms) of the collected worms from those with EPGs over 2,000 meant that there were many lost worms. Therefore the observed number of the worms was the minimum confirmed burden of infection in humans.

Although the number of recovered worms varied even in each range of EPGs, we found a moderate positive correlation between EPGs and number of worms from the human subjects (Fig. 2, *r* = 0.681, *P*<0.001). It is quite reasonable and well-known that EPGs correlate with the worm burden. This correlation was confirmed already in human opisthorchiasis [8]. This is the first report to provide the worm data of *C. sinensis* from human hosts in correlation with EPGs.

In the present study, the proportion for individuals in the range of EPG 1–1,000 was 62.0% of egg-positive subjects, but their worm recovery rate was only 9.7%. Hong et al. [6] suggested that the KK and FES method is highly sensitive for diagnosis of clonorchiasis. Based upon the present findings, the sensitivity of the worm collection method is too low to apply for diagnosis of clonorchiasis. Of course the worm collection is too complicated to be used for routine diagnosis. It is unable to use the worm collection as a diagnostic method of clonorchiasis.

We expected that it was reliable to conjecture the number of infected worms according to EPGs. However, it is impossible to figure exact number of infected worms from humans because we do not know numbers of lost worms, and EPG counting by the KK method is a crude method. Thus, numbers of *C. sinensis* in the present study were minimum confirmed figures from the infected individuals. Based on these limitations, we focused on two subjects who discharged 114 and 106 worms because their numbers of recovered worm were higher than those of most subjects. With data of them, it is roughly estimated that at least 110 worms are infected in a human body with EPGs around 3,000. No one knows how many worms were lost exactly but the 114 and 106 worms from the two patients were regarded as almost close to a real number. If the two patients passed roughly 150 g of feces in a day, total number of eggs per day from them might be 450,000. If the 110 worms produce 450,000 eggs per day, a worm produces about 4,090 eggs. This amount of eggs per one *C. sinensis* was the first estimation from human hosts. This egg productivity per day is the highest estimation compared with those of various laboratory animals [9]. The human is a suitable host for *C. sinensis*, and thus the worms would produce maximum eggs in a human body.

Median EPG per worm (EPGPW) in the present study was higher than that in human opisthorchiasis (514 versus 200) [8]. However, because of a large variation in our study, it is difficult to compare EPGPW between human clonorchiasis and opisthorchiasis. In a study on *O. viverrini*, a theoretical equation between EPGs and worm number was figured through accurate recovery of worms by autopsy [22]. The gradual decrease of EPWPD according to the increase of recovered worm counts may depend on declining fecundity by high density of worms in the intrahepatic bile ducts [22]. We were not able to calculate EPWPD as well as EPGPW due to small sample size and loss of worms in the present study. Further, the aged worms of *C. sinensis* may produce fewer eggs than young ones, and this may be another causative factor of relatively loose correlation between egg counts and worm burden in the present study as compared with analysis in human opisthorchiasis [23].

Chai et al. [18] reported that most of the tried patients passed the adult worms of *O. viverrini* and maximum 315 worms were collected from a man in Laos. Comparing the worm collection data between *C. sinensis* and *O. viverrini*, it is regarded more difficult to collect *C. sinensis* than *O. viverrini* from humans. The difficulty may originate from their size difference, since *C. sinensis* is bigger than *O. viverrini*.

It was reported that the cure rate in human clonorchiasis was 83.0% after the first praziquantel treatment and also converted to 100% by the second treatment [24]. Since we collected worms by one treatment in the present study, it is necessary to monitor uncured cases.

In conclusion, EPGs are well correlated with number of worms in human clonorchiasis. It is estimated that at least 110 worms are infected in a human body with EPGs around 3,000, and EPWPD is around 4,000. However, worm collection from humans is very limited and complicated although it is the only non-invasive method to obtain information of worms in human clonorchiasis.
